# Impact of Pathology Review in Adverse Histological Characteristics and pT Stages of Upper Tract Urothelial Cancer in a Multicenter Study

**DOI:** 10.3389/fonc.2021.757359

**Published:** 2021-11-25

**Authors:** Chia-Hui Chang, Wen-Jeng Wu, Hsiang-Ying Lee, Chih-Hung Lin, Chung-Tai Yue, Yuan-Hong Jiang, Yu-Khun Lee, Kuan Hsun Huang, Yao Chou Tsai

**Affiliations:** ^1^ Division of Endocrine and Metabolism, Department of Internal Medicine, Taipei Tzu Chi Hospital, The Buddhist Medical Foundation, New Taipei City, Taiwan; ^2^ Department of Urology, Kaohsiung Medical University Hospital, Kaohsiung, Taiwan; ^3^ Department of Urology, School of Medicine, College of Medicine, Kaohsiung Medical University, Kaohsiung, Taiwan; ^4^ Graduate Institute of Clinical Medicine, College of Medicine, Kaohsiung Medical University, Kaohsiung, Taiwan; ^5^ Department of Pathology, Kaohsiung Municipal Hsiaokang Hospital, Kaohsiung, Taiwan; ^6^ Department of Pathology, College of Medicine, Kaohsiung Medical University, Kaohsiung, Taiwan; ^7^ Department of Anatomic Pathology, Taipei Tzu Chi Hospital, Buddhist Tzu Chi Medical Foundation, New Taipei City, Taiwan; ^8^ School of Medicine, Tzu Chi University, Hualien, Taiwan; ^9^ Department of Urology, Hualien Tzu Chi Hospital, Buddhist Tzu Chi Medical Foundation and Tzu Chi University, Hualien, Taiwan; ^10^ Division of Urology, Department of Surgery, Dalin Tzuchi Hospital, The Buddhist Tzu Chi Medical Foundation, Chiayi, Taiwan; ^11^ Department of Urology, School of Medicine, College of Medicine, Taipei Medical University, Taipei, Taiwan; ^12^ Department of Urology, Taipei Medical University Hospital, Taipei Medical University, Taipei City, Taiwan; ^13^ TMU Research Center of Urology and Kidney (TMU-RCUK), Taipei Medical University, Taipei, Taiwan

**Keywords:** upper urinary tract cancer, variant histology, pathology review, intratubular spread, interobserver agreement

## Abstract

**Purpose:**

Pathology reviews for upper urinary tract cancer (UTUC) remained scarce in the literature. Here, we reported the interobserver variation among the review and local pathologies of featured histologic characteristics for UTUC.

**Methods:**

Patients who underwent definitive surgical treatments for UTUC were retrospectively reviewed for eligibility of pathology review. In the Taiwan UTUC Collaboration cohort, 212 cases were reviewed, of which 154 cases were eligible for pathology review. Agreement between original pathology and review pathology was measured by the total percentage of agreement and by simple kappa statistics. The prognostic impact was analyzed by the Cox regression model with the estimation of hazard ratios (HR) and 95% confidence intervals.

**Results:**

There were 80 women and 74 men enrolled in this study, and the median age at treatment was 71.7 years. The agreement is moderate agreement for surgical margin status (87.7%; κ = 0.61), tumor grade (82.5%; κ = 0.43), tumor invasiveness (76.6%; κ = 0.45), lymphovascular invasion (70.8%; κ = 0.42) and T stage (67.5%; κ = 0.52). The interobserver agreements for perineural invasion and variant histology identification were slight. Kaplan–Meier analysis for disease-free survival revealed comparable results in local and review pathology for localized (Tis, Ta, T1–2) or advanced T stage (T3–4).

**Conclusions:**

Pathology review of UTUC had minimal impact on clinical practice based on current available disease treatment guidelines. However, significant interobserver variations were observed in featured adverse histopathological characters.

## Introduction

Histopathological analysis of radical nephroureterectomy (RNU) specimens provides important information in evaluation of the prognosis for upper urinary tract urothelial cancer (UTUC). According to NCCN guidelines of UTUC, histological information is crucial in determining surgical approaches (RNU or kidney sparing management) and the necessity of systemic adjuvant treatments after RNU.

To achieve an accurate histopathological staging, several consensuses have been reached to help in handling specimens, identifying reliable histopathological techniques, and providing a standardized pathological report (1, 2). Unlike bladder urothelial cancer, due to the complexity of the upper urinary tract system, delicate gross examination with adequate sampling and understanding of the microanatomy of the pelvicalyceal and ureter system are crucial for accurate staging. Several technical factors, such as poor fixation of friable tumors and processing artifacts, would lead to difficulties in accurate staging and thus intra- and interobserver bias (1).

Pathology review has been recommended in multicenter cancer studies, especially in rare cancers, to enhance the consistency in the diagnosis, classification, and pathological staging of tumors, which is very helpful and important in data analysis (3). Significant interobserver variations have been reported in bladder urothelial cancer (4–7). Prior studies in reviewing the histology of bladder cancer revealed significant discrepancies in tumor grade and stage among local and central pathologies, and these disagreements were more commonly found in high-risk cases (4–6). Review of UTUC histology was rarely discussed in the literature, basically only focusing on variant histology which is an adverse histologic character and is commonly under-recognized and reported in a prior study (7).

Whether pathology review for those important histological factors, such as tumor grading, staging, and surgical margin status, of UTUC will have an impact on clinical practice, patient outcome, or multicenter study largely remained unknown. In addition, due to the rarity of UTUC, the histological review analysis for UTUC remained extremely scarce in the literature. Here, we reported the interobserver variation among the review and local pathologists for pathological stages, surgical margin status, and featured histologic characteristics for UTUC. In addition, these histologic factors were analyzed for their prognostic impact on survival outcomes according to review and local pathologies.

## Material and Methods

### Data Source

This UTUC prospective registry database was conducted by the Taiwan UTUC Collaboration Group. The Taiwan UTUC prospective database is a multicenter internet-based registry, which enrolled 212 cases from 12 hospitals in Taiwan since June 2018. This study was reviewed and approved by the institutional review board (IRB no. 06-X34-105). Informed consent was obtained from all participants. The study protocols and methods were carried out in accordance with relevant guidelines and regulations.

### Patients and Specimens

Patients who underwent UTUC surgical treatments, including radical nephroureterectomy (RNU), endoscopic management, and segmental resection, were retrospectively reviewed for eligibility for histological review. In this cohort, 212 cases were reviewed, of which 154 cases were eligible for specimen review. The exclusion criteria for pathology review were cases without a definite surgical treatment (RNU, endoscopic management or segmental resection) and having no accessible full set of pathology slide for review. Of those 58 cases excluded for pathology review, 39 of them did not undergo definite surgical treatment and 19 of them had no accessible full set of pathology slides for review. Those who were eligible for review were sent a full set of slides for pathology review. The definition of full set of slides is those sections examined by the local institutional pathologist which were all sent for review.

### Histological Review

The histological review was carried out by a single pathologist who is the recommended consultant genitourinary pathologist of the Taiwan Society of Pathology using a standardized histological report format, which was approved by the Taiwan Pathology Society based on the AJCC TNM staging system and the principles of pathology management for urothelial cancer in NCCN guidelines. The central pathological reviewer was blind to the detailed initial pathological diagnosis of the local pathologist except gross description and number of sections of specimen, because the reviewer was unable to access the information from the original specimens. The median number of the reviewed full set slides was 9 with an inter-quartile range of 8 to 12 slides. The histological diagnosis and staging of UTUC specimens were based on the version 9 American Joint Committee on Cancer (AJCC) tumor-node-metastasis (TNM) staging system, and a histological grade was made according to the 2015 WHO/ISUP recommendation grading system. The histological diagnosis of UTUC variants has been accepted by the uropathological community, and the diagnostic criteria were described in the WHO classification of tumors (8). In addition to staging, tumor grading, and variant subtypes, the appearance of tumor configuration, presence of stromal invasion, lymphovascular invasion (LVI), perineural invasion (PNI), and surgical margin invasion were recorded. The histological finding of intra-tubular spread is retrograde spread of urothelial cancer within the renal tubules, which is commonly an *in situ* process (Ta/Tis) and can be mistaken as parenchymal invasion (1). This histological character was not included in the checklist of local contributing pathologists and therefore was only presented in review pathology but not in local.

### Follow-up

The follow-up schedule for patients was every 3–6 months in the first year then every 6–12 months thereafter. Chest radiography and cross-sectional imaging [computer tomography (CT) or/and magnetic resonance images (MRI)] were used to determine recurrence/progression-free statuses. Ureteroscopy was used to detect upper-tract recurrence after endoscopic management or segmental resection. UTUC recurrence was defined as local recurrence of tumor bed, regional lymph nodes, or distant metastasis. The primary end point of this trial is disease-free survival (DFS) defined as the time from surgical treatment to first disease recurrence or death for any cause.

### Statistical Analysis

Demographic and clinicopathological differences between groups were compared using Pearson chi-square for categorical variables. The T stage, tumor grading, and surgical margin status were vital histological findings, which have a profound impact on disease-related outcomes and were therefore selected for detailed agreement analysis. In addition, due to the limited time of follow-up for the current prospective cohort which started in 2019, the disease-free interval was used to evaluate the prognostic impact of local and review pathologies. Agreement between original pathology and review pathology was measured by the total percentage of agreement and by simple kappa statistics. The margin status was only available in the RNU and segmental resection cases; thus, the endoscopically managed cases were excluded from this agreement analysis. The Kaplan–Meier estimator was used to estimate the rates of prognostic outcomes, and the survival curves were compared using the stratified log-rank test. The prognostic impact of original and review pathologies for the different parameters was analyzed by the Cox regression model with the estimation of hazard ratios (HR) and 95% confidence intervals. The Cox proportional hazard model was selected to assess the effect of prognostic outcomes by stepwise regression analysis and after adjusting for potential confounders. Statistical analyses were carried out with IBM SPSS statistical software version 26. The description of statistical methods was based on the standard format of statistical analysis of the Taiwan UTUC collaboration group.

## Results

### Baseline Characteristics

There were 80 women and 74 men enrolled in this study, and the median age at treatment was 71.7 years (inter-quartile range (IQR): 65.5–77.7). The median follow-up time was 13.22 months (IQR: 7.0–20.2). The surgical treatments were RNU in 146/154, endoscopic management in 7, and segmental resection in 1 patient (**Table 1**). One hundred and twenty-six of 146 cases managed with RUN, one of seven of endoscopically managed cases, and one case managed with segmental resection were free of disease recurrence at the end of follow-up. When comparing the histologic findings between the local pathology and the independent review pathology, a significant difference was observed in tumor stromal invasiveness, UTUC differentiation, T stage, LVI, and PNI (**Table 2**). The review pathologist identified more variant histology, LVI, and PNI than did the local pathologist. Intra-tubular spread of urothelial cancer was identified in 15.6% specimens by the review pathologist.

**Table 1:** Clinical features of upper tract urothelial cancer patients for pathology review.

Variables	N (%)
Gender	
Male	74 (48.1)
Female	80 (51.9)
Age	
<70	67 (43.5)
≥70	87 (56.5)
Location	
Renal pelvis	87 (56.5)
Ureter	60 (39.0)
Bladder cuff	3 (1.9)
renal pelvis and ureter	4 (2.6)
Surgical approach	
Nephroureterectomy	146 (94.8)
Endoscopic management	7 (4.5)
Segmental resection	1 (0.6)

**Table 2:** Histopathologic characteristics recorded by the review and local pathologist.

Variables	Local		Review		p-value
	N	%	N	%	
Grade					
Low grade	15	(9.7)	33	(21.4)	0.039
High grade	136	(88.3)	117	(76.0)	
Dysplasia	1	(0.6)	1	(0.6)	
Not mentioned	2	(1.3)	3	(1.9)	
Configuration					
Papillary	97	(63.0)	100	(64.9)	0.310
Nodular	26	(16.9)	31	(20.1)	
Infiltrating	16	(10.4)	17	(11.0)	
Flattened	5	(3.2)	3	(1.9)	
Not mentioned	10	(6.5)	3	(1.9)	
Stromal invasion					
Invasive	124	(80.5)	101	(65.6)	0.001
Non-invasive	23	(14.9)	50	(32.5)	
Not mentioned	7	(4.5)	3	(1.9)	
Differentiation					
Pure urothelial	142	(92.2)	107	(69.5)	<0.001
Squamous	7	(4.5)	9	(5.8)	
Sarcomatous	0	(0.0)	10	(6.5)	
Clear cell	0	(0.0)	4	(2.6)	
Nested variant	0	(0.0)	6	(3.9)	
Poorly differentiated	2	(1.3)	3	(1.9)	
Micropapillary	1	(0.6)	3	(1.9)	
Neuroendocrine	0	(0.0)	1	(0.6)	
Giant cell	0	(0.0)	4	(2.6)	
Mix type	0	(0.0)	5	(3.2)	
Not Available	2	(1.3)	2	(1.3)	
T stage					
Tx	3	(1.9)	0	(0.0)	0.277
Tis/Ta/T1	49	(31.8)	61	(39.6)	
T2	27	(17.5)	20	(13.0)	
T3	63	(40.9)	65	(42.2)	
T4	7	(4.5)	4	(2.6)	
Not mentioned	5	(3.2)	4	(2.6)	
Lymphovascular invasion					
Negative	104	(67.5)	86	(55.8)	0.002
Positive	42	(27.3)	67	(43.5)	
Not mentioned	8	(5.2)	1	(0.6)	
Peri-neural invasion					
Negative	107	(69.5)	132	(85.7)	<0.001
Positive	4	(2.6)	21	(13.6)	
Not mentioned	43	(27.9)	1	(0.6)	
Margin status					
Free	130	(84.4)	122	(79.2)	0.154
Not free	10	(6.5)	20	(13.0)	
Not mentioned	14	(9.1)	12	(7.8)	
Intratubular spread					
Negative	Not Available		118	(76.6)	Not Available
Positive	Not Available		24	(15.6)	
Not mentioned	Not Available		12	(7.8)	

### Level of Agreement

In total, 154 specimens were reviewed for T stage, tumor grade, and surgical margin status. Three cases that had specimens retrieved from endoscopic management were unable to make a specific T stage due to inadequate tissue. **Table 8** shows the interobserver agreement of T stage, tumor grade, and surgical margin status. According to the definition of Cohen, kappa values ≤ 0 indicate no agreement and 0.01–0.20 as slight, 0.21–0.40 as fair, 0.41–0.60 as moderate, 0.61–0.80 as substantial, and 0.81–1.00 as perfect agreement (9). Therefore, the agreement is moderate for surgical margin status (87.8%;κ = 0.52), tumor grade (82.5%; κ = 0.43), tumor invasiveness (76.6%; κ = 0.45), LVI (70.8%; κ = 0.42), and T stage (67.5%; κ = 0.52) (**Table 8**). However, the interobserver agreement for PNI and variant histology identification was slight to fair.

In the T stage, the review and local pathologists had better agreements in stage Tis/Ta/T1 and stage T3 than stage T2 and T4 (67.2/75.4% *vs*. 50/50%, percentage of review case; 83.7/77.8% *vs*. 37/28.6%, percentage of local case) (**Table 3**). In those non-agreement cases in stage, the local pathologist less frequently judged a case as lower T stage (18/154; 11.7%) than the review pathologist (24/154; 15.6%). The common disagreements in pT stage were observed in the following situations (**Supplementary Figures 1–5** and **Supplementary Material**): 1) low-grade papillary urothelial carcinoma combined with few foci of variably sized nests simulating micro-invasion, 2) high-grade ureter infiltrative urothelial carcinoma with minor foci of muscular invasion, 3) high-grade infiltrative renal pelvis urothelial carcinoma with minor foci of peri-nephritic fat invasion or small nests simulating micro-invasion, 4) high-grade infiltrating renal pelvis urothelial carcinoma with renal sinus fat invasion, and 5) renal pelvis urothelial carcinoma with intratubular spread.

**Table 3:** Interobserver variation for pathological T stage.

Review pathology	Original pathology (%*^a^*) [%*^b^*]						Total (%)
	Tx	Tis/Ta/T1	T2	T3	T4	Not mentioned	
Tis/Ta/T1	3 (4.9) [100]	41 (67.2) [83.7]	7 (11.5) [25.9]	7 (11.5) [11.1]	0 (0.0) [0.0]	3 (4.9) [60.0]	61 (39.6)
T2	0 (0.0) [0.0]	5 (25.0) [10.2]	10 (50.0) [37.0]	5 (25.0) [7.9]	0 (0.0) [0.0]	0 (0.0) [0.0]	20 (13.0)
T3	0 (0.0) [0.0]	1 (1.5) [2.0]	10 (15.4) [37.0]	49 (75.4) [77.8]	5 (7.7) [71.4]	0 (0.0) [0.0]	65 (42.2)
T4	0 (0.0) [0.0]	0 (0.0) [0.0]	0 (0.0) [0.0]	2 (50.0) [3.2]	2 (50.0) [28.6]	0 (0.0) [0.0]	4 (2.6)
Not mentioned	0 (0.0) [0.0]	2 (50.0) [4.1]	0 (0.0) [0.0]	0 (0.0) [0.0]	0 (0.0) [0.0]	2 (50.0) [40.0]	4 (2.6)
Total	3 (1.9)	49 (31.8)	27 (17.5)	63 (40.9)	7 (4.5)	5 (3.2)	154 (100)

^a^Percentage of reviewed cases.

^b^Percentage of local cases.

High-grade tumor was recorded in 122/154 (76.3%) by the review pathologist and in 140/154 (87.5%) by the local pathologist (**Table 4**). The review and local pathologists had a higher agreement in high-grade than low-grade histology (97.4% *vs*. 33.3%, percentage of review case; 83.8% *vs*. 73.3%, percentage of local case). The review pathologist more frequently recorded a tumor downgrading (21/136; 15%) than the local pathologist did (3/117; 2.5%).

**Table 4:** Interobserver variation for tumor grade.

Review pathology	Original pathology (%*^a^*) [%*^b^*]				Total (%)
	Low grade	High grade	Dysplasia	Not mentioned	
Low grade	11 (33.3) [73.3]	21 (63.6) [15.4]	0 (0.0) [0.0]	1(3.0) [50.0]	33 (21.4)
High grade	3 (2.6) [20.0]	114 (97.4) [83.8]	0 (0.0) [0.0]	0 (0.0) [0.0]	117 (76.0)
Dysplasia	0 (0.0) [0.0]	0 (0.0) [0.0]	1 (100) [100]	0 (0.0) [0.0]	1 (0.6)
Not mentioned	1 (33.3) [6.7]	1 (33.3) [0.7]	0 (0.0) [0.0]	1 (33.3) [50.0]	3 (1.9)
Total	15 (9.7)	136 (88.3)	1 (0.6)	2 (1.3)	154 (100)

^a^Percentage of reviewed cases.

^b^Percentage of local cases.

Surgical margin involvement was identified in 19/147 (12.9%) by the review pathologist and in 10/147 (6.8%) by the local pathologist (**Table 5**). Negative margins that were judged by the local pathologist were recorded as positive involvement by the review pathologist in 10/130 (7.7%) cases. There was only 1/122 (0.8%) that was recorded as a negative margin by the review pathologist, which was recorded as positive by the local pathologist.

**Table 5:** Interobserver variation for surgical margin status.

Review pathology	Original pathology (%^a^) [%^b^]			Total (%)
	Free	Not free	Not mentioned	
Free	117 (95.9) [90.0]	1 (0.8) [10.0]	4 (3.3) [57.1]	122 (83.0)
Not free	10 (52.6) [7.7]	9 (47.4) [90.0]	0 (0.0) [0.0]	19 (12.9)
Not mentioned	3 (50.0) [2.3]	0 (0.0) [0.0]	3 (50.0) [42.9]	6 (4.1)
Total	130 (88.4)	10 (6.8)	7 (4.8)	147 (100)

^a^Percentage of reviewed cases.

^b^Percentage of local cases.

### Prognostic Impact: Review Pathology *vs*. Local Pathology

**Figure 1:**
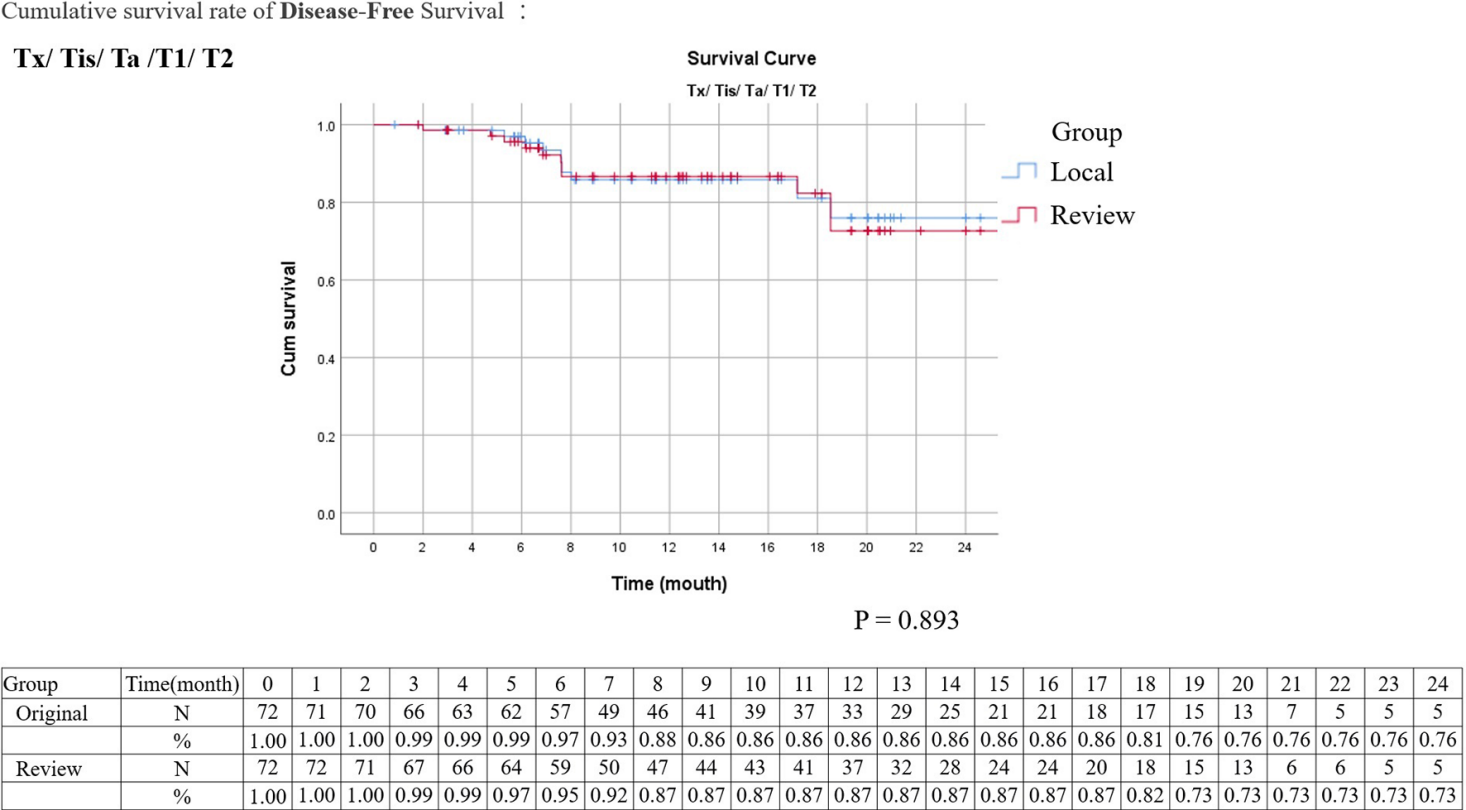
Kaplan–Meier survival curves of disease-free survival stratified by original or review pathology in localized upper tract urothelial cancers (survival curves were created and analyzed by SPSS software version 26).

**Figure 2:**
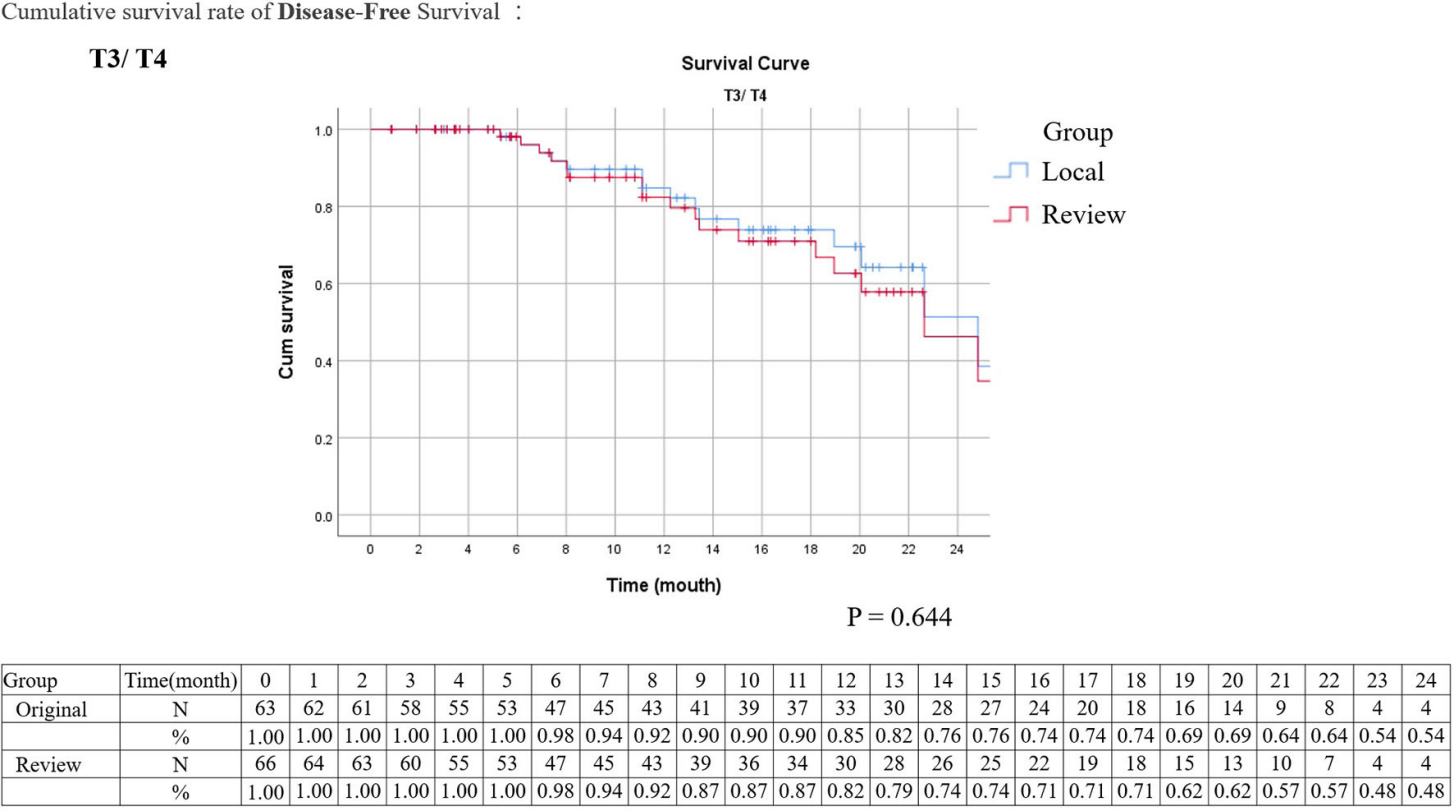
Kaplan–Meier survival curves of disease-free survival stratified by original or review pathology in advanced upper tract urothelial cancers (survival curves were created and analyzed by SPSS software version 26).

Kaplan–Meier analysis for disease-free survival revealed comparable results in localized T stage (Tis, Ta, T1–2) and advanced T stage (T3–4) after surgical treatment (**Figures 1**, **2**). The hazard ratio (HR) of the T stage by review pathology was not significant in univariable analysis, but a T4 disease by review pathology was a significant prognostic factor after adjusting for histologic confounders (HR = 0.036, 95% CI 0.002–0.840, p = 0.039) in multivariable analysis. The HR of T stage by local pathology was identified as a significant prognostic factor in univariable analysis but was excluded in multivariable analysis after a stepwise selection of independent histologic variables.

The HR of surgical margin status by review and local pathologies were both significant prognostic factors in univariate and multivariate analyses (HR = 4.15, 95% CI 1.41–12.21, p = 0.010; HR = 12.45, 95% CI 3.14–49.36, p < 0.001, respectively) (**Tables 6**, **7**). The HR of intratubular spread recorded in review pathology was a significant independent prognostic factor for disease-free survival. The HRs of tumor grade, stroma invasion status, LVI, PNI, and concomitant variant histology were not significant for review and local pathology in multivariable analysis.

**Table 6:** Agreements between review and local pathology.

Variables	Overall agreement (%)	Simple kappa (95% CI)
Grade	82.5	0.431 (0.265, 0.596)
Stroma invasion	76.6	0.447 (0.316, 0.577)
Lymphovascular invasion	70.8	0.420 (0.288, 0.552)
Peri-neural invasion	62.3	0.056 (-0.040, 0.152)
T stage	67.5	0.520 (0.418, 0.622)
Margin status	87.8	0.520 (0.419, 0.799)
Differentiation (pure urothelial or variant)	72.1	0.168 (0.029, 0.307)

**Table 7:** Comparative univariate disease-free survival analysis of upper tract urothelial cancer patients according to local and review pathology.

Univariate analysis	HR (95% CI)	p-value	Univariate analysis	Local		Review	
				HR (95% CI)	p-value	HR (95% CI)	p-value
Gender			Intratubular spread	Not Available			
Male	1		Negative			1	
Female	0.441 (0.196, 0.996)	0.049^*^	Positive			2.639 (1.075, 6.481)	0.034^*^
Age			Grade				
<70	1		Low grade	1		1	
≥70	1.514 (0.686, 3.343)	0.305	High grade	0.582 (0.218, 1.555)	0.280	1.560 (0.534, 4.555)	0.416
Location			Dysplasia	0.000 (0.000),	0.981	0.000 (0.000),	0.983
Renal pelvis	1		Stroma invasion				
Ureter	0.465 (0.183, 1.182)	0.108	Non-invasive	1		1	
Bladder cuff	4.223 (0.961, 18.553)	0.056	Invasive	1.533 (0.455, 5.163)	0.491	1.345 (0.561, 3.227)	0.507
Renal pelvis and ureter	2.148 (0.281, 16.397)	0.461	Lymphovascular invasion				
Surgical approach			Negative	1		1	
Nephroureterectomy	1		Positive	2.768 (1.173, 6.531)	0.020^*^	1.921 (0.862, 4.283)	0.110
Endoscopic	6.396 (2.492, 16.418)	<0.001^**^	Perineural invasion	Not Available			
Segmental resection	0.000 (0.000),	0.982	Negative			1	
			Positive			3.443 (1.471, 8.059)	0.004^**^
			T stage				
			Tis/Ta/T1/T2	1		1	
			T3	1.464 (0.588, 3.646)	0.413	1.988 (0.868, 4.551)	0.104
			T4	3.605 (1.079, 12.041)	0.037^*^	1.534 (0.193, 12.19)	0.686
			Tx	1.549 (0.191, 12.581)	0.682		
			Margin status				
			Free	1		1	
			Not free	4.856 (1.680, 14.037)	0.004^**^	5.557 (2.110, 14.63)	0.001^**^
			Differentiation				
			Pure UC	1		1	
			UC with variant	4.259 (1.672, 10.846)	0.002^**^	1.772 (0.790, 3.973)	0.165

CI, confidence interval; HR, hazard ratio; DFS, disease-free survival.

^*^ < 0.05. ^**^ < 0.01.

## Discussion

The tumor grading and pathological staging are vital decision-making factors in the management of UTUC according to NCCN clinical practice guidelines (10). Presence of high-grade tumor or parenchymal invasion in renal pelvis is recommended for neoadjuvant chemotherapy and RNU but not endoscopic management. Presence of high-grade tumor in the ureter is not feasible for elective endoscopic management. Presence of advanced pathological T stage is recommended for adjuvant chemotherapy following surgery. In addition, presence of surgical margin invasion is associated with more UTUC-related death; hence, adjuvant chemotherapy is recommended following RNU (11, 12). However, interobserver variation for staging parameters of UTUC has never been investigated. In the experienced hands of the review pathologists of the current study, more a positive surgical margin was revealed, and overgrading or overstaging was less commonly observed. Regarding the agreement rate, the interobserver agreements for tumor grading and pT stage were moderate with a simple kappa value between 0.41 and 0.6 (**Tables 3**, **4**, **8**). The interobserver agreement for surgical margin status was even better as 87.8% (kappa value: 0.52). In addition, Kaplan–Meier analysis for disease-free survival also revealed comparable survival curves in localized T stage and advanced T stage between local and review pathology. To the best of our knowledge, this is the first study clarifying the interobserver variations in critical pathological staging factors of UTUC, which filled the knowledge gap on the impacts of pathology review in UTUC. Although interobserver variations clearly existed in tumor grading and pT stages, these variations were of little clinical significance with minimal impact to clinical practice.

**Table 8:** Comparative multivariate disease-free survival analysis of upper tract urothelial cancer.

Multivariate analysis	Local		Review	
	HR (95% CI)	p-value	HR (95% CI)	p-value
Age				
<70	1		1	
≥70	3.229 (1.052, 9.913)	0.040^*^	7.856 (2.014, 30.643)	0.003^**^
Gender				
Male			1	
Female			0.283 (0.101, 0.793)	0.016^*^
Intratubular spread				
Negative			1	
Positive			11.592 (2.890, 46.494)	0.001^**^
T stage				
Tis/Ta/T1/T2			1	
T3			1.434 (0.346, 5.946)	0.620
T4			0.036 (0.002, 0.840)	0.039^*^
Margin status				
Free	1		1	
Not free	4.149 (1.410, 12.209)	0.010^*^	12.454 (3.142, 49.362)	<0.001^**^

CI, confidence interval; HR, hazard ratio; DFS, disease-free survival.

^*^ < 0.05. ^**^ < 0.01.

Histological identification of adverse histologic factors other than pT stage and tumor grading is also important in cancer treatment planning after surgery and critical for multicenter trials. The tumor configuration, LVI, PNI, and variant histology were linked to clinical outcomes of UTUC and therefore important for outcome analysis in the multicenter study and development of the outcome prediction model. In the current pathology review, we found comparable records between local and review pathology in tumor configuration (p = 0.31), whereas minor interobserver variations did exist in identification of stromal invasion and LVI with moderate interobserver agreement. However, the diversities in identification of PNI and variant histology were significant among local and review pathologists (kappa value: 0.05 and 0.17, respectively). The interobserver variations also led to dis-concordance in prognostic impact analysis (**Table 6**). In the univariable analysis of local pathology, LVI and variant histology were significant adverse factors for DFS. Conversely, PNI was the only significant adverse histologic factor in the univariable analysis of review pathology. Although genitourinary pathologists in Taiwan followed the same training program, specimen manipulation protocol, diagnostic criteria, standardized report template, and peer review system in each local institution, significant interobserver variations are still the challenge of multicenter studies that should be properly addressed with referral for pathology review for poorly recognized histologic features, such as variant histology and PNI. In addition, there is a clear trend that experienced review consultant pathologists are more confident and likely to report a LVI, PNI, and variant histology than local pathologists.

Although UTUC is distinct from bladder urothelial cancer in terms of invasiveness, anatomical location, and treatment strategies, they shared several similar histologic features useful for study reference. A pathology review of bladder urothelial cancer has been reported to have a major report (diagnosis, stage, and/or grade) change in one-third of cases of biopsy or tumor resection specimens (6). In addition, Lee and their colleagues found that the pathology review for transurethral bladder tumor resection specimens would alter treatment plans in about one-third of cases (13). Another similar study based on biopsied bladder UC specimens revealed pathologic changes in 27.4% of cases and potential treatment plan changes in 15.3% of cases (14). In the current pathology review for UTUC, we found a change in pT stage in 31% and tumor grading change in 17.5% of reviewed cases. In addition, we found major treatment recommendation changes regarding adjuvant systemic chemotherapy in 20/154 (13%) of cases with 6 (3.9%) of them up-staged to pT stage ≥2 and 14 (9%) of them down-staged to pT <2. Unlike prior bladder UC studies, UTUC specimens were almost reviewed after RNU; hence, major surgical treatment changes were not feasible in the current study design. Due to the high complexity of RNU specimens, agreements between local and review pathologies theoretically could not be easier or better than prior bladder UC series. Based on our findings, although major treatment recommendation changes were not common in the reviewed pathology of UTUC, the impact of these recommendation changes deserved longer follow-up in clarifying their impact on clinical outcomes.

### UTUC With Variant Histology

UTUC with a variant histopathology is not uncommon, accounting for 9%~34% among historical UTUC case series that managed with RNU (15–18). A pathology review for UTUC with variant histology has not been reported in the literature. However, a pathology review for bladder UC revealed significant interobserver variations in reporting variant histology (14). Upon review by an expert genitourinary pathologist for bladder UC, the agreement rate of pathology review was as low as 46% and multiple-variant subtypes would coexist at differing proportions in one specimen (14). In the current UTUC cohort, about 30% of cases had at least one variant subtype based on review pathology, which is comparable to historical series. Thirty-three (21.4%) of 154 cases had a change in variant subtype upon pathology review with an interobserver agreement rate of 72.1% (kappa value: 0.168). With respect to the impact of interobserver variation on DFS, UTUC with a variant histology was a significant adverse factor [(HR = 4.26, 95% CI 1.67–10.84, p = 0.002] in univariable analysis of a local pathology report; however, the effect was not observed in univariable analysis of a review pathology report (HR = 1.77, 95% CI 0.79–3.97, p = 0.165). In multivariable analysis, UTUC with variant histology was not a significant risk factor for DFS both in local and review pathology reports (**Table 7**). Possible explanations to the above phenomenon were limited sample size, limited duration of follow-up, and/or identification of more low-risk variant histology in the review pathology. Therefore, prior study has advocated that reporting the amount of each variant as a percentage of the total lesion could help in clarifying which variant subtype in what percentage would adversely affect outcomes of patients with variant histology (19). In brief, due to slight interobserver agreement in variant histology, a pathology review by an expert genitourinary pathologist and a report by the amount of each variant subtype are mandatory in clarifying the role of variant histology in UTUC.

### Intratubular Spread of UTUC

Intratubular spread of UTUC is another rarely recognized histologic feature of UTUC, which was never included in the standard report template of the Taiwan Pathology Society and also barely reported in the literature. This retrograde spread of urothelial cancer within renal tubules was proposed as an *in situ* lesion (pTa or Tis); however, sometimes it could be mistaken as parenchymal invasion due to its peculiar histologic presentation (20, 21). Sarungbam and colleagues found that intratubular spread is a common feature in renal pelvis UTUC, which accounted for 31.5% of cases in their report (21). They also found that intratubular spread was associated with a variety of histological features, which is crucial for accurate staging. The impact of intratubular spread on clinical outcome has never been reported in the literature. In the current study, we identified 24/154 (15.6%) cases harboring a histologic feature of intratubular spread by review pathologists. With a median follow-up of 13.2 months, presence of intratubular spread of UTUC was an independent adverse risk factor of DFS (HR = 11.59, 95% CI 2.89–46.49, p = 0.001). To the best of our knowledge, this is the first time that a special histologic feature of intratubular spread in UTUC was identified as an adverse histologic factor of cancer outcome thus far. Therefore, a further prospective multicenter study with pathology review is mandatory in clarifying the role of intratubular spread in pathological staging and patient outcomes.

### Limitations

Several limitations were observed in the current study. First, the current study was derived from a newly developed prospective multicenter clinical database; therefore, the power of this study was limited by small case number and short duration of follow-up which could possibly underestimate the impact of pathology review on oncological outcomes. Second, although the current study accounted for interobserver bias in pathology, the intra-observer reliability in reporting pathology was not the scope of the current study and not analyzed. Third, lacking of standard templates in reporting specific variant histology, PNI, and intratubular spread inevitably introduced bias in the reported results. For limited case number and similar good clinical outcomes in pTa, T1, and Tis staging, these cases were grouped as one group in agreement analysis. Although this change would have little impact on outcome analysis, interobserver dis-concordance could be underestimated in this subgroup. Fourth, another limitation is that the review pathologist could not have full access to the original specimen. To minimize this limitation, the gross description, number of sections of specimen, and full set of pathology slides were provided to the review pathologist. Finally, significant diversities among the review and local pathologies were observed in featured adverse histologic findings such as LVI, PNI, variant histology, and intra-tubular spread which might have an impact on patient outcome but not listed as treatment change factors in the current available treatment guidelines. Therefore, the pathology review in UTUC may have a minimal impact for clinical practice but could impact the outcomes in multicenter trials.

## Conclusion

Although a pathology review of UTUC by experienced pathologists had minimal impact on clinical practice based on current available disease treatment guidelines, significant interobserver variations and impact were observed in featured adverse histopathological characters, such as LVI and variant histology. Therefore, these adverse histologic features should be properly addressed with referral for pathology review in multicenter trials of UTUC. In addition, intra-tubular spread of UTUC is a potential risk factor of disease recurrence and hence deserved prospective multicenter study in clarifying the impact of this under-recognized histologic feature.

## Data Availability Statement

The original contributions presented in the study are included in the article/**Supplementary Material**. Further inquiries can be directed to the corresponding author.

## Ethics Statement

The studies involving human participants were reviewed and approved by the Taipei Tzu Chi General Hospital IRB no. 06-X34-105. The patients/participants provided their written informed consent to participate in this study.

## Author Contributions

All authors listed have made a substantial, direct, and intellectual contribution to the work and approved it for publication.

## Conflict of Interest

The authors declare that the research was conducted in the absence of any commercial or financial relationships that could be construed as a potential conflict of interest.

The reviewer C-HH declared a shared affiliation, with no collaboration, with one of the authors, YT, to the handling editor at the time of the review.

## Publisher’s Note

All claims expressed in this article are solely those of the authors and do not necessarily represent those of their affiliated organizations, or those of the publisher, the editors and the reviewers. Any product that may be evaluated in this article, or claim that may be made by its manufacturer, is not guaranteed or endorsed by the publisher.
