# Supplementary material for: Impact of Pathology Review in Adverse Histological Characteristics and pT Stages of Upper Tract Urothelial Cancer in a Multicenter Study
Source: Front Oncol. 2021 Nov 25;11:757359. doi: 10.3389/fonc.2021.757359 (PMC8655678; doi:10.3389/fonc.2021.757359)
Supplement: Supplementary file 1 [file DataSheet_1.docx]

**Supplementary material:**

*
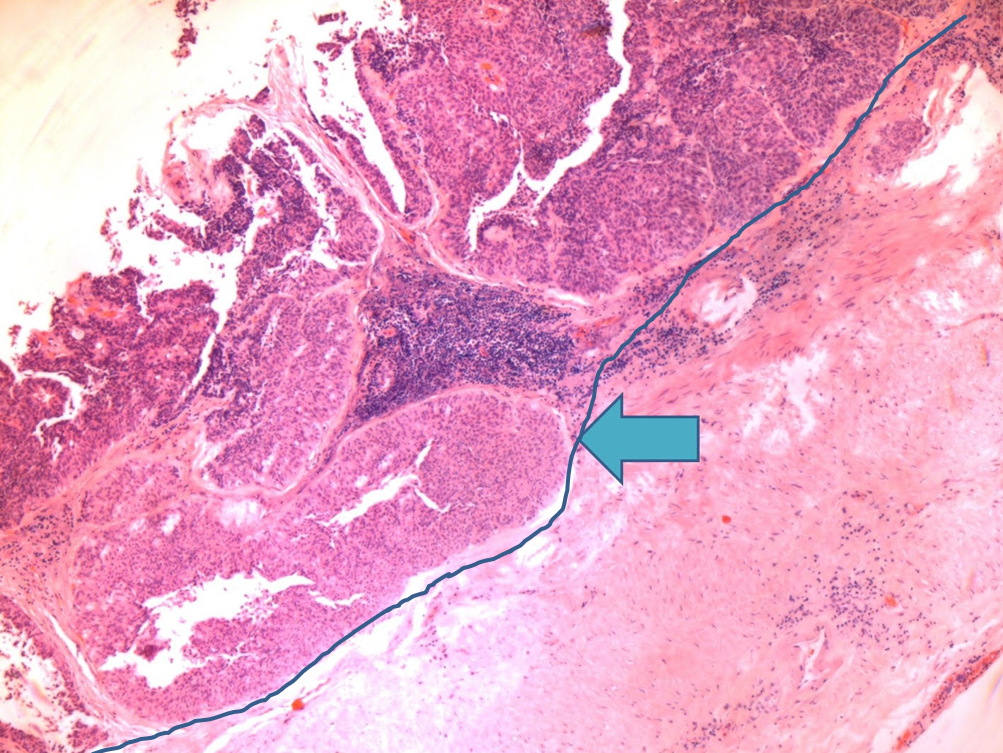
*

*Suppl. Fig.1: In this typical example of exophytic low grade papillary urothelial carcinoma, few foci of variably-sized invaginated nests leaded to a misinterpretation of stromal invasion by the local pathologist. This is a folded papillary growth and common finding in GU pathological practice. It is helpful to draw an imaginary line according to the basal membrane of non-invasive tumor nearby.*

*
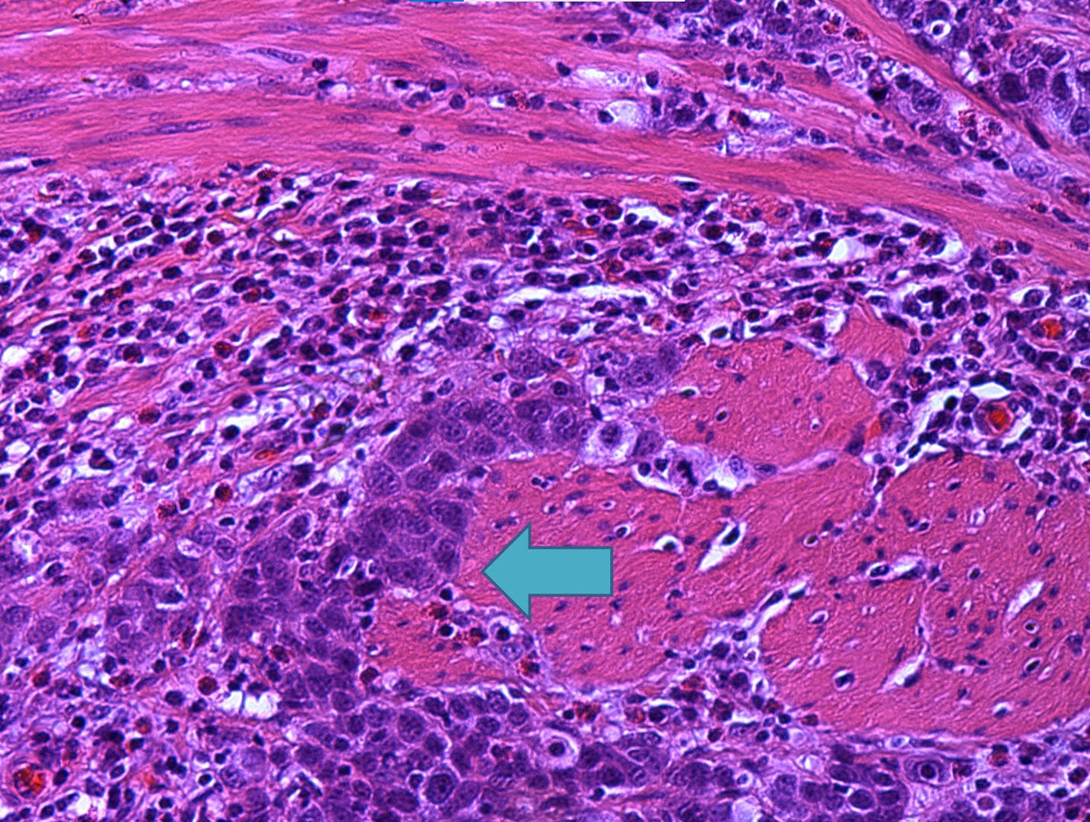
*

*Suppl. Fig.2: In this example of High grade infiltrative urothelial carcinoma of ureter, minor foci of muscular invasion were missed by the local pathologist. It is prudent to scan the entire areas of tumor-stroma border meticulously to avoid overlook. Also, the depth of invasion should be correlated with preoperative image studies and gross findings. An incorrect assessment of muscle-invasive tumor may lead to under-treatment of the patient.*

*
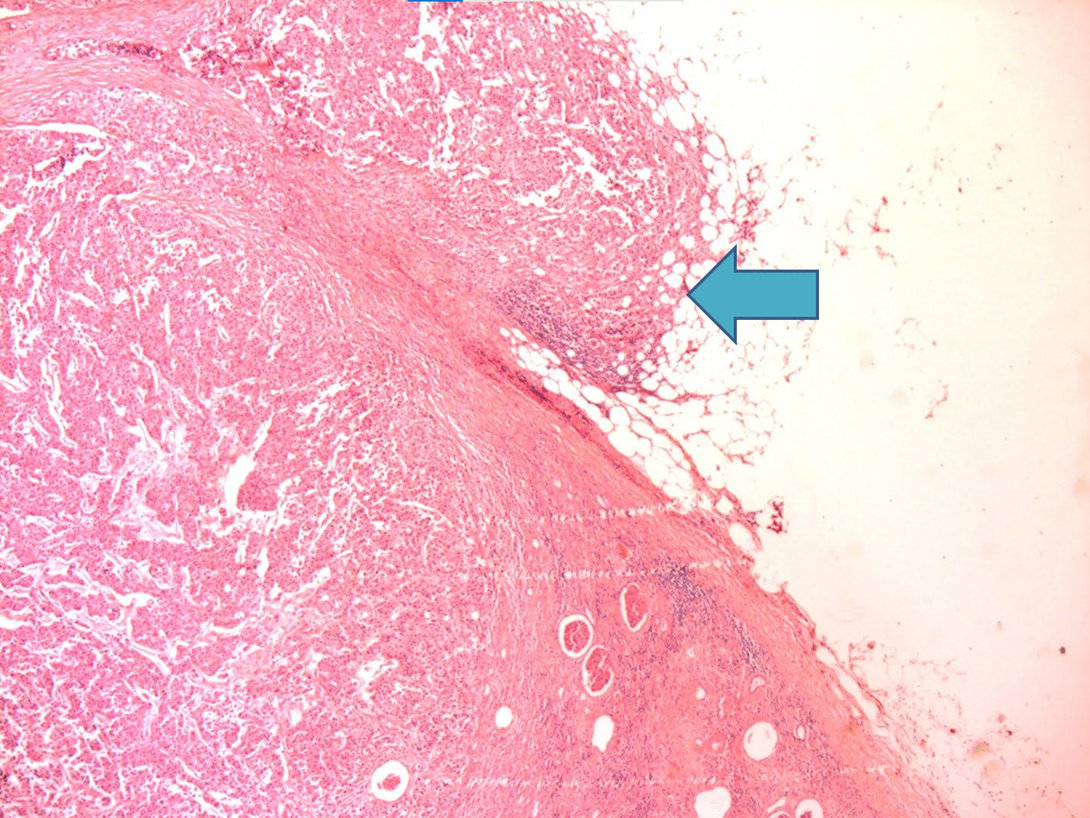
*

*Suppl. Fig.3: A high grade infiltrative urothelial carcinoma of renal pelvis. Minor foci of perinephritic fat invasion were over-looked by the local pathologist. It is helpful to scan the entire renal capsule meticulously to avoid overlook. Also, the extent of invasion should be correlated with preoperative image studies and gross findings. An misinterpretation of T4 tumor as T3 may lead to under-treatment of the patient.*

*
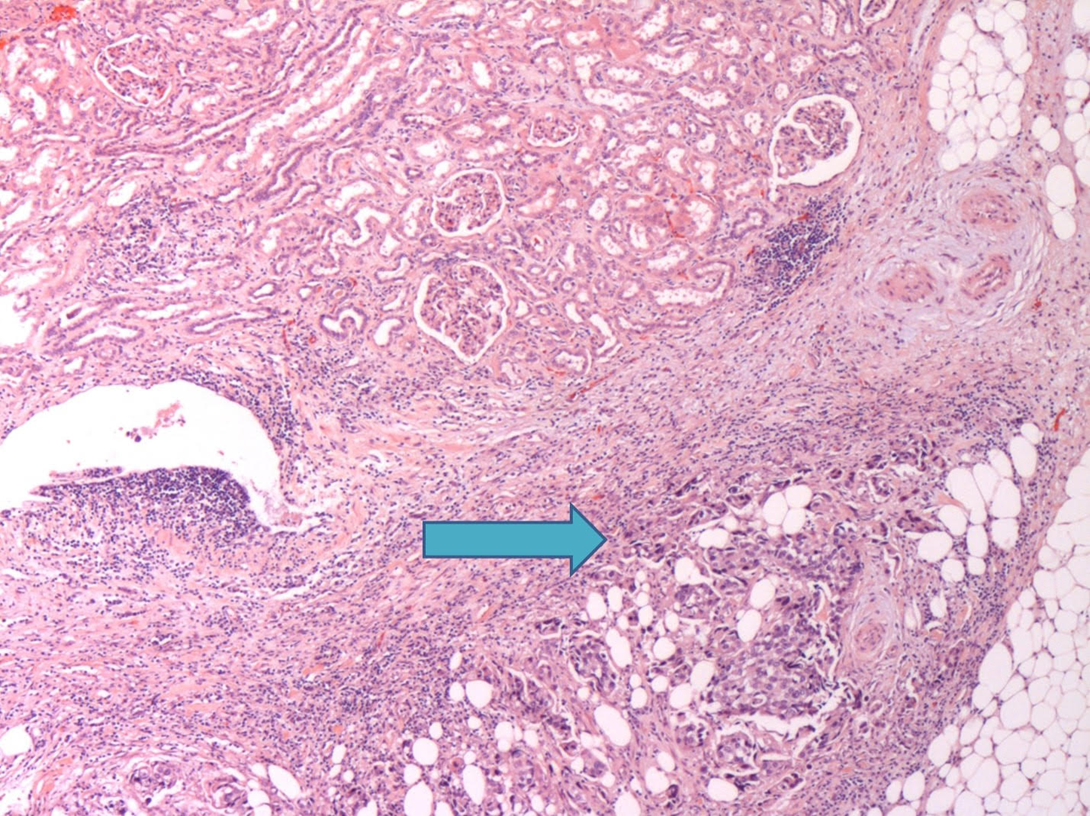
*

*Suppl. Fig.4: In this example of high grade infiltrating urothelial carcinoma of renal pelvis, renal sinus fat invasion (T3) was misinterpreted as invasion of perinephritic fat (T4). Prominent Renal sinus fat and renal parenchyma invasion were identified. Invasion through the entire thickness of renal parenchymal must be observed to assign a renal pelvis tumor as T4. Also, the extent of invasion should be correlated with preoperative image studies and gross findings. An misinterpretation of T3 tumor as T4 may lead to over-treatment of the patient.*

*
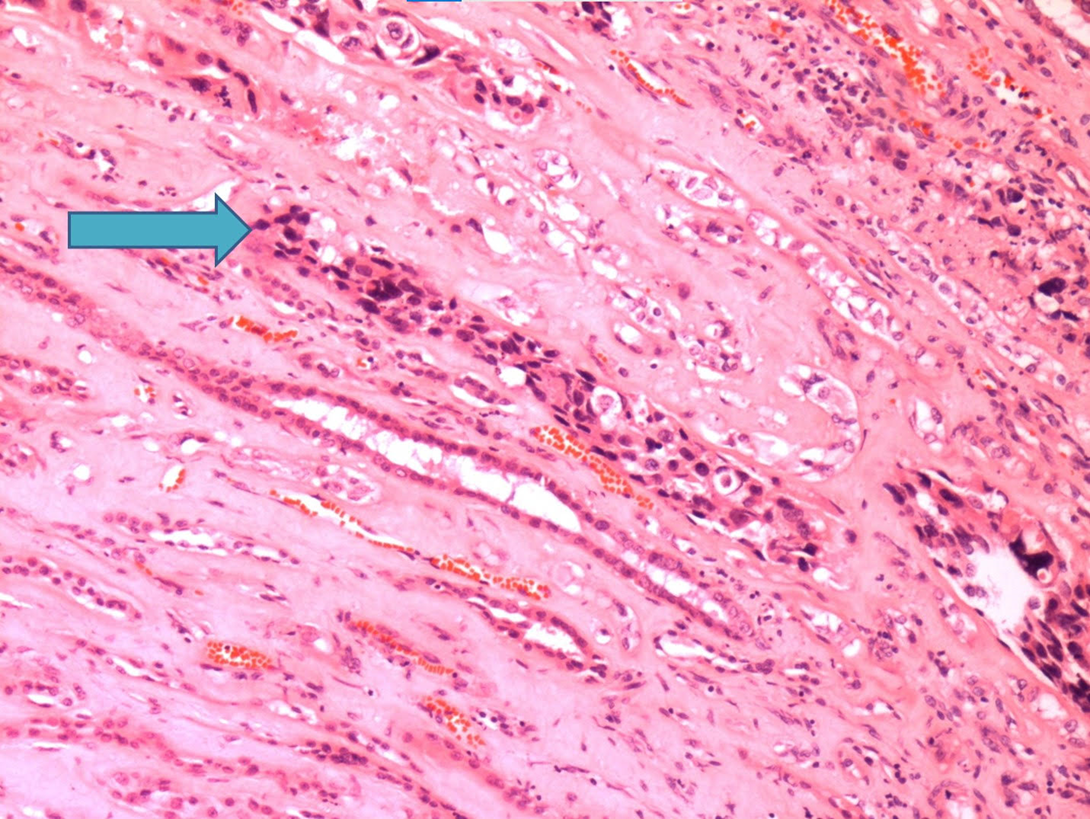
*

*Suppl. Fig.5: High-grade infiltrating urothelial carcinoma of renal pelvis. Prominent intratubular spread was identified. Intratubular spread must be distinguished from stromal invasion of renal parenchyma. In comparison to the renal tubules nearby, the renal tubular shape is maintained without distortion in the case of intratubular spread. Furthermore, desmoplastic stroma is not present in the case of intratubular spread.*
